# Intramolecular Interactions in Derivatives of Uracil Tautomers

**DOI:** 10.3390/molecules27217240

**Published:** 2022-10-25

**Authors:** Paweł A. Wieczorkiewicz, Tadeusz M. Krygowski, Halina Szatylowicz

**Affiliations:** 1 Faculty of Chemistry, Warsaw University of Technology, Noakowskiego 3, 00-664 Warsaw, Poland; 2 Department of Chemistry, University of Warsaw, Pasteura 1, 02-093 Warsaw, Poland

**Keywords:** substituent effect, solvent effect, hydrogen bond, tautomers, nitro group, amino group

## Abstract

The influence of solvents on intramolecular interactions in 5- or 6-substituted nitro and amino derivatives of six tautomeric forms of uracil was investigated. For this purpose, the density functional theory (B97-D3/aug-cc-pVDZ) calculations were performed in ten environments (1 > ε > 109) using the polarizable continuum model (PCM) of solvation. The substituents were characterized by electronic (charge of the substituent active region, cSAR) and geometric parameters. Intramolecular interactions between non-covalently bonded atoms were investigated using the theory of atoms in molecules (AIM) and the non-covalent interaction index (NCI) method, which allowed discussion of possible interactions between the substituents and N/NH endocyclic as well as =O/−OH exocyclic groups. The nitro group was more electron-withdrawing in the 5 than in the 6 position, while the opposite effect was observed in the case of electron donation of the amino group. These properties of both groups were enhanced in polar solvents; the enhancement depended on the *ortho* interactions. Substitution or solvation did not change tautomeric preferences of uracil significantly. However, the formation of a strong NO∙∙∙HO intramolecular hydrogen bond in the 5-NO_2_ derivative stabilized the dienol tautomer from +17.9 (unsubstituted) to +5.4 kcal/mol (substituted, energy relative to the most stable diketo tautomer).

## 1. Introduction

Uracil is a common and naturally occurring pyrimidine derivative. The best known occurrences of uracil are probably nucleic acids, as it is one of the five bases of the nucleic acid. In RNA, uracil forms a complementary base pair with adenine, while its 5-methylated derivative, called thymine, is an equivalent base in DNA [1]. Uracil and its derivatives have also found applications in other branches of biochemistry. For example, 5-fluorouracil is used in treatment of several cancer types by chemotherapy [2,3], while 5-bromo and iodo uracil derivatives are studied as radiosensitizers for radiotherapy [4,5,6,7]. In 2013, a computational study of various 5-substituted uracil derivatives (X = CN, SCN, NCS, NCO, OCN, SH, N_3_, NO_2_) was performed in order to identify the most suitable radiosensitizers for experimental studies [8]. The most promising derivatives with high electron affinities, 5-(N-Trifluoromethylcarboxy)aminouracil [9], 5-thiocyanatouracil [10] and 5-selenocyanatouracil [11], were synthesized. Among them, 5-thiocyanatouracil has already been tested against prostate cancer cells with promising results [12]. Some uracil derivatives show antifungal and antimicrobial properties, whereas others act as inhibitors of specific enzymes [13]. On the other hand, some of them are mutagenic, for example, 5-hydroxyuracil [14]. An interesting novel class of compounds that are derived from nucleic acid base molecules, including uracil, are ferrocene-like complexes in which the nitrogen base molecule is attached to one of the cyclopentadienyl ligands [15]. It is a relatively new class of compounds that may find applications in pharmacy, biology and electrochemistry.

An important issue regarding nucleic acid bases is tautomerism. Each of the bases can exist in several forms that differ in the position of the labile hydrogen atom. In general, one of these forms is more stable than the others, and most of the molecules exist in that form [16,17,18]. For this reason, RNA and DNA base pairs are built only from N9H tautomer of purine bases and N1H of pyrimidine bases [1]. However, relative stability of the tautomers can significantly change upon oxidation, reduction [17], substitution of the nucleobase [19,20], polarity of the environment [17] and even interaction with a metal cation [21,22]. Tautomerism of nucleobases is of interest in knowledge of biochemical processes. Importantly, it has been proposed that the existence of rare tautomeric forms can cause mutations of genetic code recorded in the DNA or alter functions performed by different variants of RNA [23,24,25,26,27]. Therefore, much effort has been put into studying the properties of uracil and its tautomers, including both theoretical and experimental studies ([16,28] and references therein). As mentioned above, various uracil derivatives are used or currently being studied for medical applications, where they are introduced into the human body. For this reason, investigating which factors can affect the tautomeric equilibria of uracil (and how) is a relevant research topic.

**Figure 1:**
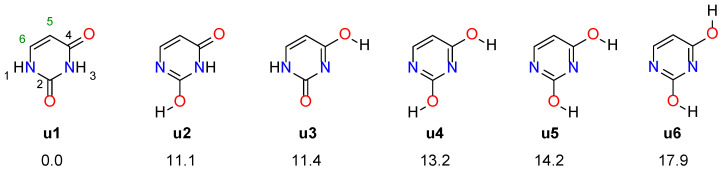
Four most stable tautomers of uracil (**u1**–**u4**) and two rotamers of **u4** (**u5**, **u6**). The numbers given below are their relative energies in kcal/mol.

Uracil consists of a pyrimidine ring and two attached −OH groups at the 2 and 4 positions. However, the most stable tautomeric form has both hydrogen atoms of the −OH groups attached to the nitrogen atoms in the pyrimidine ring. The four most stable uracil tautomers (**u1**–**u4**) and their two rotamers (**u5**, **u6**), along with their relative stabilities, are shown in Figure 1. Based on calorimetric experiments [29], it was found that the dienol form is 20 ± 10 kcal/mol less stable than **u1**, while **u3** by 19 ± 6 kcal/mol. In addition, both diketo (**u1**) and keto-enol tautomers (**u2**, **u3**) were identified using the dispersed fluorescence spectra, although the precise structure of the latter was not determined [30]. The most stable keto-enol tautomer was estimated to have about 9.6 kcal/mol higher energy than the diketo form (**u1**).

The aim of the research is to investigate both the intramolecular interactions in uracil derivatives and their sensitivity to solvent change, as well as their ability to change tautomeric preferences. Similar studies on adenine and purine derivatives were recently carried out [31,32]; our computational results were in agreement with the experimental NMR data of 8-halopurines obtained by other groups [19,20].

For this study, we selected the 5- and 6-substituted nitro and amino derivatives of the six tautomeric forms of uracil (Figure 1). The nitro and amino groups represent model electron-withdrawing and electron-donating substituents, respectively. In addition, the nitro group rotated by 90 degrees from the plane of the ring was taken into account. This group interacts with the substituted system only inductively, as opposed to the planar NO_2_ group, which acts through induction and resonance.

Two substitution positions, 5 and 6, differ in through-space *ortho* interactions and through-bond interactions with endocyclic N atoms/NH groups as well as −OH/=O groups. In position 5, depending on the tautomeric form, the substituent can interact through-space with the C4=O or C4−OH group. In turn, the substituent in position 6 can interact through-space with the N or NH group in the 1 position. Regarding the through-space interactions, in some cases, formation of an intramolecular hydrogen bond is possible. Thus, the question arises whether it can alter tautomeric preferences.

Regarding the through-bond interactions, the 5 position is *meta*-related towards two endocyclic N/NH groups and *ortho*- and *para*-related towards two exocyclic −OH/=O groups. Conversely, the 6 position is *meta*-related to the −OH/=O and *ortho*- and *para*-related towards N/NH. Here, it is important to mention that in pyrimidines, the position of the substituent in relation to the endocyclic N atoms has a profound influence on the substituent–substituted system interaction, which affects the electron-withdrawing/donating strength of substituents. This topic is discussed in our recent paper [33].

It should be emphasized that the −OH and =O groups have opposite electronic properties: the −OH group is an electron-donating substituent, whereas =O is an electron-withdrawing substituent. Therefore, the tautomeric form should be important for the intramolecular interactions in uracil derivatives.

## 2. Methodology

Quantum chemical DFT calculations [34,35] were performed in the Gaussian 16 program [36]. We used the B97-D3/aug-cc-pVDZ method, in accordance with our recent research regarding purine and adenine derivatives [31,32,37]. The optimized geometries correspond to the minima on the potential energy surface since no imaginary vibrational frequencies were found. In the constrained optimization cases, i.e., systems with the NO_2_ group rotated by 90 degrees, one imaginary frequency corresponding to the rotation along the C-N bond was found.

**Figure 2:**
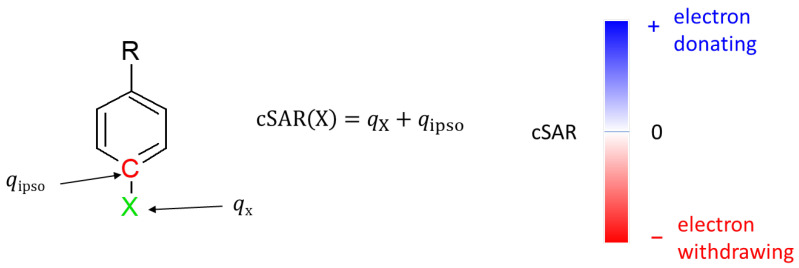
Definition of cSAR and interpretation of its value. *q*_X_ is the sum of atomic charges of all atoms forming a substituent X, while *q*_ipso_ is the atomic charge at the *ipso* atom.

Electronic properties of substituents were evaluated using the charge of the substituent active region (cSAR) parameter [38,39]. Its definition is presented in Figure 2. Positive cSAR values indicate the deficit of electrons in the substituent active region, i.e., the substituent is electron-donating. Negative values represent an excess of electrons in the active region of the substituent, indicating its electron-withdrawing properties. To allow comparison with our other results, the atomic charges used to calculate cSAR were obtained by the Hirshfeld method [40].

In order to study solvent effects, the IEF-PCM implicit model of solvation was used [41,42,43]. Calculations were performed in ten media, listed in Table 1 along with their dielectric constants. It should be mentioned that the PCM has been used many times in computational studies of nucleic acid bases [6,17,19]. In the AT and GC base pairs, the molecular geometries obtained with the PCM were in good agreement with the experimental data and the calculations using the H_2_O microsolvation model [44].

Analysis of electron density using the atoms in molecules (AIM) theory [45] was performed in the AIMAII program [46]. The main goal of this analysis was the search for possible bond critical points (BCPs) of non-covalent intramolecular interactions. When such a BCP was present, we estimated the interaction energy according to the formula of Afonin et al. (Equation (1)) [47], derived from the Espinosa equation [48].

(1) $$E_{\mathrm{HB}}=0.277\;\cdot \;V_{\mathrm{BCP}}+0.450$$

where *V*_BCP_ is the potential energy density (in kcal ∙ mol^−1^ ∙ bohr^−3^) at the BCP. This equation can be applied to OH⋯O, OH⋯N, OH⋯halogen, NH⋯O, NH⋯N, CH⋯O, CH⋯N and CH⋯halogen interactions.

Intramolecular interactions between non-covalently bonded atoms were also investigated using the non-covalent interaction index (NCI) method [49]. The nature of a given interaction was assigned and color-coded according to the value of sgn(λ_2_) ∙ *ρ*(r), where λ_2_ is the second eigenvalue of the Hessian matrix of electron density (*ρ*(r)). Points on reduced density gradient isosurfaces with a value of sgn(λ_2_)∙*ρ*(r) > 0 indicate non-bonding (steric) contacts (in red), with sgn(λ_2_)∙*ρ*(r)~0 indicating weakly attractive interactions (e.g., van der Waals, in green) and sgn(λ_2_)∙*ρ*(r) < 0 indicating strongly attractive interactions (e.g., hydrogen and halogen bonding, in blue). For more information on the NCI analysis, see Johnson et al. [49]. In our case, NCI calculations were performed in Multiwfn 3.8 software [50] and the visualization in the VMD program [51].

## 3. Results and Discussion

### 3.1. Electronic Properties of Substituents

The raw data generated in this study and used in statistical analyses are available in the Supplementary Materials. Table 2 presents the cSAR values of the substituents in all studied systems. In the case of amino derivatives, the NH_2_ substituent in position 6 has more than twice, in the cSAR scale, stronger electron-donating properties than in position 5. In nitro derivatives, the NO_2_ group in position 5 is more electron-withdrawing than in position 6. Therefore, the substitution position, i.e., the position in relation to the nitrogen atoms in the ring, has a decisive influence on the properties of the substituent. In contrast, the effect of the tautomeric form of uracil is clearly less significant. It is also worth noting that in polar solvents, the characteristic properties of both NO_2_ and NH_2_ groups are enhanced, as shown by the difference between cSAR(X) values in the water and gas phase (Δ).

In 5-NH_2_ derivatives, electron-donating strength of the amino group decreases in the sequence: **u2** > **u5**~**u4**~**u1** > **u3** > **u6**. The clearly lower cSAR(X) for **u6** is a consequence of the rotation of the NH_2_ group by 90 degrees and the formation of the hydrogen bond, H_2_N∙∙∙HO, which is discussed in more detail later in the paper. In this case, the large influence of the solvent on the cSAR(NH_2_) value is due to the rotation of the NH_2_ group to more planar conformation with respect to the ring in polar solvents. This strengthens the resonance effect. 

In 6-NH_2_ derivatives, electron-donating strength of the amino group decreases in the sequence: **u3** > **u1**~**u5** > **u6**~**u4** > **u2**. Two systems containing the NH endocyclic group at the *ortho* position, **u3** and **u1**, have the greatest electron-donating properties. An interesting difference is present between the **u5** form and its rotamers: **u4** and **u6**. Among them, the highest cSAR(NH_2_) value and the lowest Δ occur in **u5**, where the two OH groups are facing in the same direction. When they are in opposite directions, as in **u4** and **u6**, the value of cSAR(X) is lower, while Δ is higher. This may be due to the differences in the dipole moments in these two cases, as the conformation of the OH groups has a significant impact on the value and direction of molecular dipole moment (Table S1). By far the strongest solvent effect on cSAR(X) among the 6-NH_2_ derivatives occurs in **u1** and **u3** (highest Δ). These systems also have the highest values of the dipole moment (Table S1). All cSAR(NH_2_) values in 5-NH_2_ derivatives are lower than in aniline (0.094), while in 6-NH_2_ they are higher.

Generally, in all 5-NO_2_ tautomers, the NO_2_ group is withdrawing electrons more strongly than in nitrobenzene, where the cSAR(X) is higher, −0.140. Its rotation by 90 degree increases cSAR(NO_2_) by about 0.4 units. The only exception is **u6**, where a decrease in cSAR is observed; however, this is caused by the hydrogen bonding between the NO_2_ and *ortho* OH groups. In 5-NO_2_ systems, electron-withdrawing strength of the nitro group decreases in the sequence: **u3** > **u4**~**u5** > **u2** > **u1** > **u6**. The systems with the strongest electron-withdrawing NO_2_ groups (**u3**, **u4** and **u5**) have an electron-donating OH group in the *ortho* position, but its hydrogen atom is directed to the endocyclic N atom, so that NO∙∙∙OH interaction can be expected. When NO∙∙∙HO interaction is present (5-NO_2_ **u6**), the electron-withdrawing ability of the NO_2_ group is the weakest. Again, the greatest variability of cSAR(X) due to solvation occurs in the derivatives with the highest values of the dipole moments (**u1** and **u2**).

**Figure 3:**
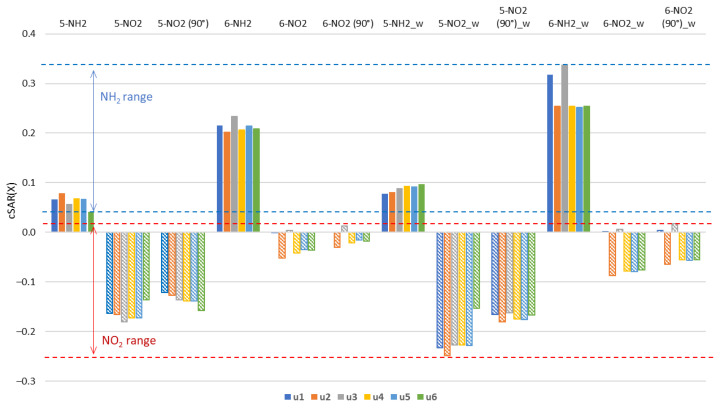
Values of cSAR(X) (in *e*) for X = NH_2_, NO_2_ groups in the gas phase and in the aqueous solution (_w). The 90° in parentheses indicates nitro derivatives where 90° rotation around the CN bond was forced.

In the 6-NO_2_ derivatives, the cSAR(NO_2_) values are high, indicating weak electron-withdrawing properties. This is caused by the disturbance of the resonance interactions by ring nitrogen atoms in *ortho* and *para* positions. Weak resonance is also evidenced by a smaller increase in cSAR due to the rotation of NO_2_ by 90° as compared to the 5-NO_2_ derivatives. This increase is by about 0.2 units, with the exception of **u1** and **u3** where cSAR(NO_2_) is positive and its change due to rotation is smaller. Electron-withdrawing strength decreases in the sequence: **u2** > **u4** > **u6**~**u5** > **u1** > **u3**. The loss of electron-withdrawing properties (cSAR close to 0.0) of the 6-NO_2_ group occurs in the **u1** and **u3** derivatives, where the NH group is in the *ortho* position. Thus, apart from the relative position of the endo N atoms and the substituent, the NO∙∙∙HN through-space interaction has an effect as well. The summary of the cSAR analysis in the form of a bar chart is shown in Figure 3.

In most cases, the dependences of cSAR(X) on 1/ε are well approximated by a linear function. The parameters of resulting cSAR (X) = *a*∙(1/ε) + *b* functions are summarized in Table 3. The slope value, *a*, informs about the sensitivity of the electronic properties of the substituent in a given derivative to the solvent effect. In general, except for **u6** 5-NH_2_, large absolute values of the coefficient occur in systems with a large dipole moment, and small ones in systems with a small dipole moment (Table 3 and Table S1). In 6-substituted systems (6-NO_2_ and 6-NH_2_), the values of *a* in **u1** and **u3** (*ortho* NH) clearly differ from other tautomers (*ortho* N). This can be attributed to the influence of *ortho* interactions with endocylic N/NH groups. It can be concluded that the repulsive *ortho* interaction, NH∙∙∙HN for 6-NH_2_ and NO∙∙∙N for 6-NO_2_, causes high sensitivity of the substituent properties to the solvent effect, whereas the attractive interaction causes low sensitivity. A similar effect was observed in adenine and purine derivatives [29,30].

**Figure 4:**
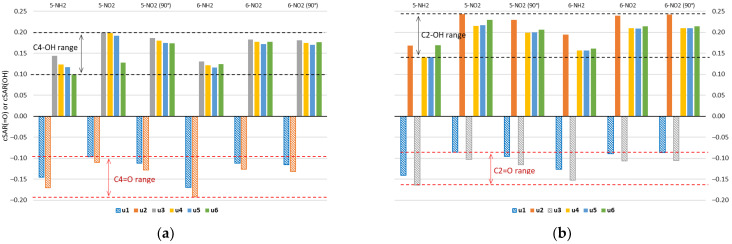
Values of cSAR(=O) or cSAR(OH) (in *e*) for two groups in (**a**) C4 and (**b**) C2 position of uracil molecule. Data for NH_2_- and NO_2_-substituted uracil derivatives in the gas phase.

Properties of the =O/−OH groups of all studied forms of uracil, quantified by cSAR, are shown in Figure 4. Negative values correspond to the electron-withdrawing =O group, whereas positive values to the electron-donating −OH. Both the interactions with the substituent and the type of tautomer can affect the electron-donating (−OH) or -withdrawing (=O) properties of these groups. The electron-withdrawing properties of the =O groups are greater in the amino derivatives than in the nitro derivatives, which is shown by the more negative cSAR(=O) values in the 5-NH_2_ and 6-NH_2_ derivatives. In turn, the electron-donating properties of the −OH groups are greater in the nitro than in the amino derivatives. This is due to charge transfer between groups with opposite electronic properties. Global ranges of variation of cSAR are 0.143 for the =O group and 0.107 for the −OH group. The ranges for the =O group in C4 and C2 positions are 0.096 and 0.078, respectively, while the average values are −0.134 for C4 and −0.115 for C2. In the case of the −OH group, the ranges are 0.099 for C4 and 0.103 for C2 positions; the average values are 0.159 for C4 and 0.199 for C2. Thus, the characteristic electronic properties of the −OH group are on average stronger in the C2 position, while those of the =O group are stronger in the C4 position. Stronger electronic properties are accompanied by higher ranges of their variability. 

The C2 position of the uracil ring is double *ortho* with respect to the two *endo* N/NH atoms/groups, while the C4 position is *ortho* and *para*. So, two electronegative atoms in the *ortho* position of the −OH group might enhance its electron-donating properties, while diminishing the electron-withdrawing by the =O group. A similar effect of *ortho* N atoms on the substituent properties was observed in our recent studies on nitro and amino derivatives of pyridine, pyrimidine, pyrazine and triazine [33]. 

### 3.2. Geometry

**Figure 5:**
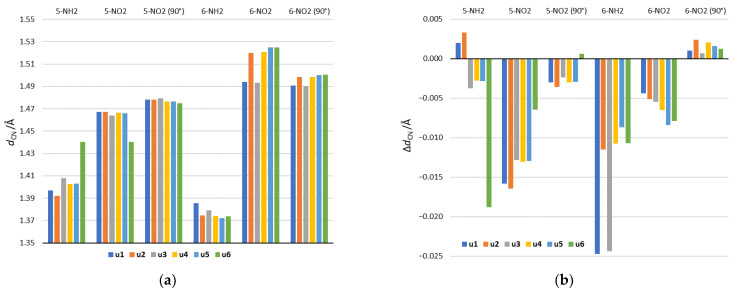
(**a**) The lengths of the CN bonds, connecting the substituent and substituted system in the gas phase and (**b**) differences between their lengths in formamide (the most polar solvent) and in the gas phase. Positive values of Δ*d*_CN_ indicate longer bond in formamide than in the gas phase, while negative values indicate shorter.

Analysis of geometry will be focused on the lengths of CN bonds connecting the NO_2_ and NH_2_ substituents and the substituted system. As shown in Figure 5a, they vary depending on the substitution position and the tautomeric form. In the case of 5-NH_2_ derivatives, the shortest CN bond occurs in the **u2** tautomer and the longest in **u6**. The **u2** tautomer is also characterized by the highest electron-donating strength of the NH_2_ group among 5-NH_2_ derivatives (Table 2 and Figure 3). In the case of the **u6** tautomer in the gas phase, the NH_2_ group is rotated by 90 degrees in order to form a H_2_N∙∙∙HO hydrogen bond with the OH group in the *ortho* position. This is accompanied by a significant extension of the CN bond, which reaches the length observed for the 5-NO_2_ group in u6. In 6-NH_2_ derivatives, CN bonds are shorter than in 5-NH_2_, which is connected with the strong electron-donating 6-NH_2_ group. A slightly longer bond relative to other tautomers occurs in **u1** and **u3**. This may be due to the presence of the NH group in the *ortho* position resulting in NH∙∙∙HN steric interaction. 

In NO_2_ derivatives, shorter CN bonds are found in 5-NO_2_ than in 6-NO_2_ systems. This is in line with the electron-withdrawing strength of the 5-NO_2_ and 6-NO_2_ groups. In position 5, the shortest bond occurs in **u6**, where a strong NO∙∙∙HO hydrogen bond is formed, while the second shortest is in **u3**, in which the NO_2_ group has the strongest electron-accepting properties among all systems. For 6-NO_2_ tautomers, clearly the shortest bonds occur in **u1** and **u3**, where the NH group is in the *ortho* position. This results from the attractive NO∙∙∙HN interaction.

The rotation of the NO_2_ group in 5-NO_2_ derivatives causes the elongation of CN bonds, which is related to the disturbance of the resonance effect of the NO_2_ group. The largest elongation occurs in the u6 5-NO_2_ derivative. It is caused by breaking of the NO∙∙∙HO hydrogen bond as a result of NO_2_ rotation. In the 6-NO_2_ systems, in four tautomers: **u2**, **u4**, **u5** and **u6** (*ortho* N), the NO_2_ rotation clearly shortens the CN bond. This is caused by the weakening of through-space repulsive interactions with the *ortho* endocyclic N atom. Thus, the main factor determining the CN bond lengths in the 5-NO_2_ derivatives is the resonance between the NO_2_ group and the substituted system, while in the 6-NO_2_ derivatives it is the *ortho* interaction.

The solvation effect is also reflected in the CN bond lengths. Figure 5b shows the difference in CN bond lengths between the values obtained in the aqueous solution and the gas phase. In NH_2_ derivatives, a stronger solvent effect occurs in 6-NH_2_ systems, while in the case of NO_2_ derivatives, in 5-NO_2_ systems. This is connected with the greater variability of the substituent’s electronic properties in these systems (see, for example, Table 3). Thus, the bond shortening is related to an increase in the characteristic electronic properties of a given substituent, due to the increase in the solvent polarity.

### 3.3. Intramolecular Interactions between Non-Covalently Bonded Atoms 

**Figure 6:**
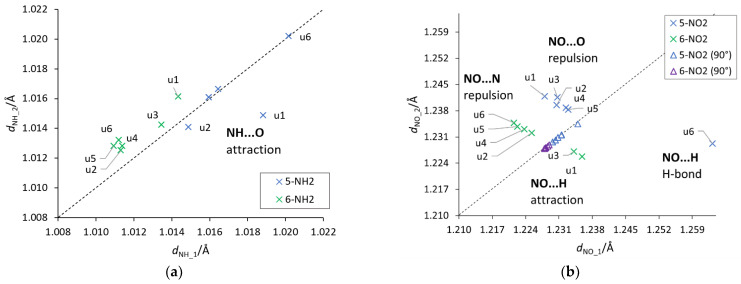
Plots between the lengths of the two (**a**) NH and (**b**) NO bonds of the NH_2_/NO_2_ groups. The dashed y = x line indicates a symmetry between the bonds. A system where asymmetry is present and the H-bond is detected (**u6** 5-NO_2_) has been marked appropriately. NH_1 and NO_1 are the bonds facing towards the lower atom numbers in the ring (for example, 4 in 5 substitution), while NH_2 and NO_2 towards higher numbers (see the atom numbering in Figure 1).

An important aspect of the interaction between the substituent and the substituted system are through-space *ortho* interactions, which in some cases could already be seen by the cSAR(X) values and CN bond lengths. In order to identify these interactions, the lengths of two NH/NO bonds of the NH_2_/NO_2_ groups were plotted against each other (Figure 6). Deviations from the equal length of these two bonds may indicate the existence of an asymmetric through-space interaction. Such plots also provide information about the attractive/repulsive nature of these interactions, based on the location of a point above or below the y = x line. 

**Figure 7:**
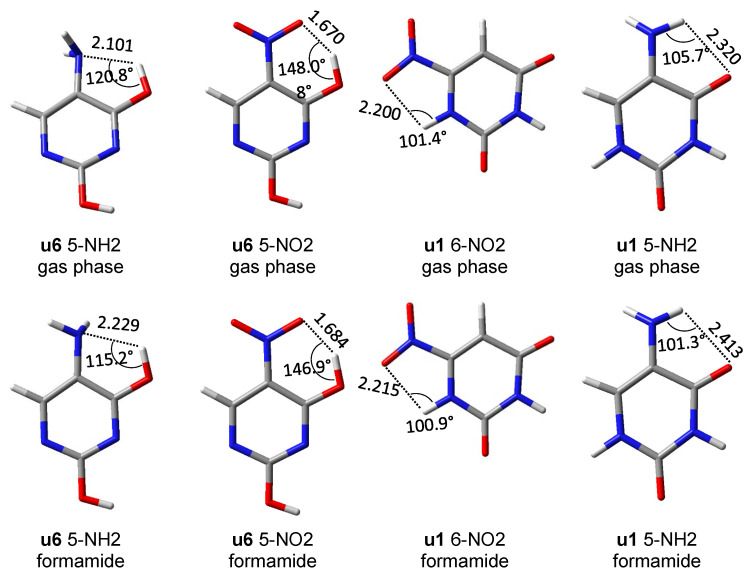
Structures of several systems, in which an interesting intramolecular interaction is present, and geometric data for this interaction (distances in Å).

First of all, it should be noticed that the asymmetry in the bond lengths of the NO_2_ group is about four times greater than that of the NH_2_ group. Moreover, for the nitro group, the obtained results indicate greater variability of interactions, but as expected in systems with rotated groups, the lengths of both NO bonds are similar. Both repulsive and attractive interactions as well as hydrogen bonds are observed. In the latter case, the systems in which the interaction meets the Koch–Popelier criteria for hydrogen bonding [52] are depicted as H-bonds in Figure 6. Only one system (in the gas phase), visible in the plot, **u6** 5-NO_2_, fulfills the criteria. Additionally, an increase in the polarity of the solvent weakens the through-space interactions—an increase in the O∙∙∙H distance and a decrease in O∙∙∙HO angle, as shown in Figure 7. An interesting system in which, despite the symmetry between NH bonds, there is a strong H-bond is **u6** 5-NH_2_. In this case, the NH_2_ group rotates by 90°, and forms a H_2_N∙∙∙HO hydrogen bond. Moreover, the NH_2_ group in the formamide solution rotates slightly towards the coplanar conformation (76.7° dihedral angle) and the H-bond is weakened. This rotation is an interesting example of competition of attractive through-space interactions and the resonance between the group and the substituted system. In the gas phase, the H-bond has a greater influence on the structure, but in the polar solvent, due to the weakening of the H-bond, stabilization by resonance forces the group to be coplanar. The structures of **u6** 5-NO_2_ and **u6** 5-NH_2_ are shown in Figure 7.

Based on the potential energy density at the critical point of each hydrogen bond, their energy was calculated from the Afonin equation (Equation (1)). For comparison, the hydrogen bond energy was also calculated using the rotational method [53], i.e., the difference between **u6** and **u5** rotamers. Both methods give similar results (Table 4), especially in the case of stronger hydrogen bonding in **u6** 5-NO_2_. 

**Figure 8:**
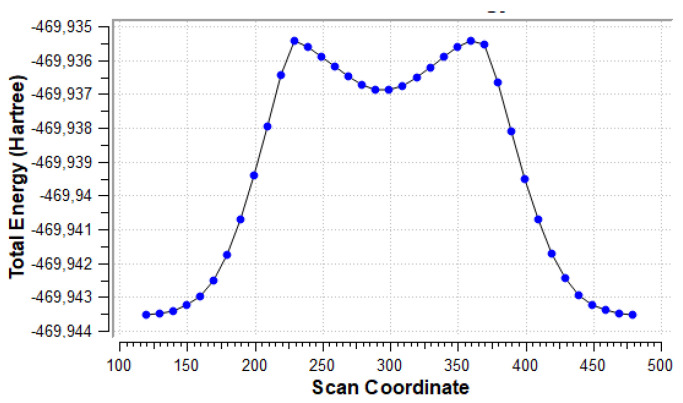
Energy scan for rotation of the NH_2_ group about the CN bond in the **u6** 5-NH_2_ system, shown in Figure 7.

Figure 8 shows the energy scan along the dihedral angle between the amino group and the uracil ring plane. The global minimum corresponds to the conformer shown in Figure 7, the minimum near scan coordinate 300 corresponds to the form rotated by 180° from the global minimum, so that NH_2_∙∙∙HO bifurcated contact is present. Two maxima correspond to forms with close NH∙∙∙HO contacts (1.956 Å). Rotational barrier height is 5.08 kcal/mol, while the difference in energy between the two minima is 4.16 kcal/mol.

**Figure 9:**
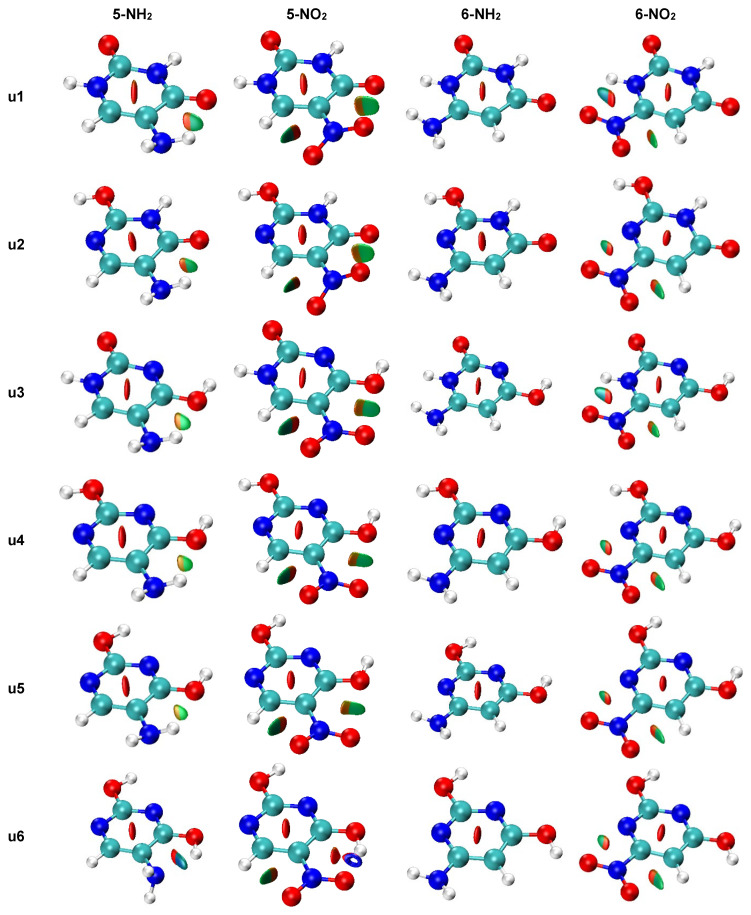
NCI plots for all studied systems (gas phase geometry). Isosurfaces correspond to the value of reduced density gradient function of 0.5. Red shading indicates non-bonding (steric) contacts, green weakly attractive interactions (e.g., van der Waals) and blue strongly attractive interactions (e.g., hydrogen bonding).

The NCI analysis, shown in Figure 9, was performed to visualize all non-covalent interactions. In most cases, only weak interactions (green-shaded isosurfaces) are present. However, in systems where the asymmetry of two NH/NO bonds (Figure 6) was high, a blue color can be noticed on the isosurfaces between the interacting atoms. This indicates a stronger attractive character of these interaction. The **u1** 5-NH_2_ system, which has the highest bond length asymmetry (Figure 6) among the amino derivatives, has very slight blue features on the isosurface between NH and =O, which indicated stronger attractive interaction than in **u2**–**u5** 5-NH_2_ systems. The intramolecular H-bond in **u6** 5-NH_2_, discussed earlier, appears as a mostly blue isosurface between H_2_N and HO. The H-bond in the **u6** 5-NO_2_ system is so strong that the NCI analysis treats it as a partially covalent interaction, as the hole is pierced through the isosurface along the H∙∙∙O line. In **u1** and **u3** 6-NO_2_ systems, some blue accents are noticeable on the isosurface corresponding to the NO∙∙∙HN contact. Bond critical points of non-covalent interactions were found only in **u6** 5-NH_2_ and **u6** 5-NO_2_. 

Interestingly, in several nitropurines, NO∙∙∙HN interactions have a bond critical point [30]. It is possible that this interaction is on the edge of being classified as H-bonding. The reasons are probably low values of O∙∙∙HN angles (105.6° in 1H 6-nitropurine vs. 101.4° in **u1** 6-NO_2_ uracil), which are close to the limit of 110° proposed by Desiraju [54], and rather high O∙∙∙H distances (2.107 Å in 1H 6-nitropurine vs. 2.200 Å in **u1** 6-NO_2_ uracil).

### 3.4. Tautomer Stability

The last section is devoted to the effects of substitution and solvation on the stability of uracil tautomers. Table 5 presents electronic energies of each system relative to the **u1** tautomer. In all cases, this tautomer remains the most stable, irrespective of substitution and solvation. Considering the 5-NO_2_ substitution, the **u6** 5-NO_2_ derivative is a particularly interesting case. Formation of a strong NO∙∙∙HO H-bond results in a large stabilization relative to the unsubstituted **u6** tautomer (by 12.5 kcal/mol). Consequently, among the 5-NO_2_ tautomers, **u6** becomes the second most stable tautomer after **u1**, despite the fact that **u6** is the least stable tautomer for unsubstituted uracil. Rotating the 5-NO_2_ group by 90 degrees and breaking the hydrogen bond increases the relative energy of **u6** by 10.4 kcal/mol and in 5-NO_2_ (90°), **u6** is again the least stable tautomer. 

In the case of 5-NH_2_ substitution, the energy difference between the **u1** and **u2** tautomers decreases compared to the unsubstituted systems, while between **u1** and others it increases. In 6-NH_2_, the relative energies are smaller than for unsubstituted systems. A noteworthy increase in stability relative to **u1** is observed for **u2**, **u4**, **u5** and **u6** tautomers (between 5 and 6 kcal/mol), while much less for **u3** (1.1 kcal/mol). In the case 6-NO_2_ tautomers, apart from **u3**, the relative energies decrease slightly, but not as much as in 6-NH_2_. In all cases, the relative energies between the **u1** tautomer and the second most stable one are above 5.4 kcal/mol; therefore, it is unlikely that substitution with NH_2_ or NO_2_ groups can significantly affect the tautomeric equilibrium. Solvation. in most cases, further increases the difference between **u1** and the other forms, as evidenced by the positive values of Δ (apart of two cases) in Table 5. The only cases where Δ is negative are the two NH_2_ derivatives of the **u3** tautomer: **u3** 5-NH_2_ (Δ = −2.2 kcal/mol) and **u3** 6-NH_2_ (Δ = −0.6 kcal/mol). 

Similarly to the cSAR (X), electronic energy can be plotted against 1/ε and relations approximated with straight lines can be obtained (Table 6). In this case, the slopes (*a*) inform about the sensitivity of the energy of a given system to the solvent effect. In most cases, the **u1** and **u3** tautomers are the most sensitive, these two tautomers have an endo NH group in the 1 position of the uracil ring. The only exception is the 6-NO_2_ substitution, where the **u2** and **u6** tautomers are most sensitive to the solvent effect. The **u4** and **u5** tautomers are in all but one case (H-bond forming **u6** 5-NO_2_) the least sensitive. In amino derivatives, the sensitivity to the solvent effect seems to be correlated with the dipole moments of the molecules, i.e., a large dipole moment is associated with a large value of *a*. However, no such relation can be observed in the case of nitro derivatives.

**Figure 10:**
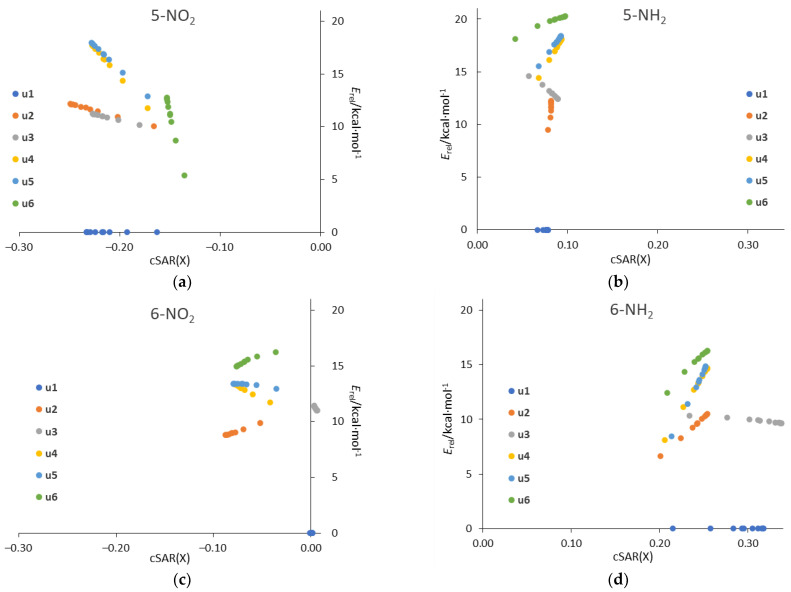
Plots of relative energy of tautomer against the cSAR(X) (in *e*) for (**a**) 5-NO_2_, (**b**) 5-NH_2_, (**c**) 6-NO_2_, (**d**) 6-NH_2_ systems in all considered solvents.

Plotting the relative energy, *E*_rel_, against the cSAR(X) for all systems in all solvents (Figure 10) reveals linearly correlated groups of points for each tautomer. The linearity comes from the fact that both *E*_rel_ and cSAR change linearly with 1/ε (see Table 3 and Table 6). The ranges on the y and x axes for particular tautomers are a visual representation of the strength of the solvent effect on *E*_rel_ and cSAR, respectively. It is clearly visible that, in general, the greatest changes in both parameters occur for the 5-NO_2_ and 6-NH_2_ derivatives.

## 4. Conclusions

This work is devoted to the influence of the substituent and solvent on the tautomeric preferences and intramolecular interactions of uracil. For this purpose, the four most stable uracil tautomers and two rotamers of the dienol form, substituted by nitro and amino groups at C5 and C6 positions in ten environments, were studied. In addition, changes in the properties of the substituents were also realized by rotating the NO_2_ group 90 degrees about the CN bond. The research was carried out using the DFT-D method and the polarizable continuum solvent model (PCM). 

In uracil derivatives, the properties of the substituents depend primarily on their position with respect to endocyclic N atoms and less on the tautomeric form. Changing the =O to −OH group in the C2 or/and C4 position has less effect on the electronic properties of the substituent (and geometry), despite their opposite electronic properties. Therefore, the relationships between the relative position of endocyclic N atoms and the substituent on its electronic properties and geometry observed in simple monosubstituted N-heterocycles (pyridine, pyrimidine, pyrazine, etc.) [33] can be applied to more complex systems, such as uracil. Thus, the NH_2_ substituent at the 6 position of uracil has more than twice (in the cSAR scale) stronger electron-donating properties than at the C5 position. In contrast, the NO_2_ group has more electron-withdrawing power in position C5 than in position C6. The characteristic properties of both NO_2_ and NH_2_ groups are enhanced in polar solvents. The strength of the solvation effects on the substituent properties depends on through-space *ortho* interactions. This has also been previously observed in purine and adenine derivatives [29,30]. 

Regarding the intramolecular interactions between non-covalently bonded atoms, both repulsive and attractive interactions, including hydrogen bonds, are observed. This is evidenced by the results of the NCI and AIM analyses and geometric parameters. Interesting hydrogen bonding interactions, NO∙∙∙HO and H_2_N∙∙∙HO (with NH_2_ rotated by 90°), were found in **u6** 5-NO_2_ and 5-NH_2_ derivatives, respectively. The NO∙∙∙HO interaction is strong and it highly stabilizes the **u6** tautomeric form of 5-NO_2_ derivative, with respect to other tautomers. The attractive interactions between the 6-NO_2_ group and endocyclic NH group (NO∙∙∙HN) are visible on the NCI plots and in the geometry data, but do not have the bond critical point. Interaction between the NH_2_ group and endocyclic N atom (NH∙∙∙N) is not detectable by any method.

The substitution of the uracil molecule, as well as the solvation effects, does not significantly alter its tautomeric preferences. This differs from what has been reported for purine and adenine derivatives, where substitution and solvation significantly affected the tautomeric equilibrium [17,19,29,30]. However, the observed decrease in the relative energy of **u6** and **u2** uracil tautomers due to the 5-NO_2_ and 6-NH_2_ substitution, respectively, may cause an increase in the amount of enol tautomers in the equilibrium mixture.

## Figures and Tables

**Table 1:** Media in which calculations were performed, and their dielectric constants, ε.

Formamide	Water	DMSO	Ethanol	Pyridine	THF	*o*-Cresol	Chloroform	Toluene	Gas Phase
108.94	78.36	46.83	24.85	12.98	7.43	6.76	4.71	2.37	1.00

**Table 2:** Values of cSAR(X) (in elementary charge units, *e*) for X = NH_2_, NO_2_ groups in the gas phase. Δ indicates a difference between the cSAR(X) values in the aqueous solution (PCM) and the gas phase.

Taut.	5-NH_2_	Δ	5-NO_2_	Δ	5-NO_2_ (90°)	Δ	6-NH_2_	Δ	6-NO_2_	Δ	6-NO_2_ (90°)	Δ
**u1**	0.067	0.011	−0.163	−0.070	−0.121	−0.045	0.215	0.102	−0.001	0.002	0.001	0.003
**u2**	0.078	0.003	−0.166	−0.082	−0.127	−0.053	0.201	0.052	−0.052	−0.035	−0.030	−0.034
**u3**	0.057	0.032	−0.180	−0.047	−0.136	−0.026	0.234	0.103	0.004	0.003	0.013	0.005
**u4**	0.068	0.025	−0.173	−0.055	−0.138	−0.037	0.206	0.048	−0.042	−0.036	−0.021	−0.034
**u5**	0.068	0.025	−0.172	−0.055	−0.139	−0.037	0.214	0.038	−0.035	−0.044	−0.015	−0.041
**u6**	0.042	0.055	−0.136	−0.018	−0.157	−0.009	0.208	0.045	−0.036	−0.040	−0.018	−0.037
range	0.037	0.052	0.045	0.064	0.036	0.044	0.032	0.065	0.056	0.047	0.043	0.046

**Table 3:** Parameters of cSAR(X) = *a*∙(1/ε) + *b* linear regressions: slopes, *a*, and determination coefficients, *R*^2^; unit cSAR(X) is *e*.

Tautomer	5-NH_2_		5-NO_2_		5-NO_2_ (90°)		6-NO_2_		6-NO_2_ (90°)		6-NH_2_	
	*a*	*R* ^2^	*a*	*R* ^2^	*a*	*R* ^2^	*a*	*R* ^2^	*a*	*R* ^2^	*a*	*R* ^2^
**u1**	−0.011	0.993	0.071	0.957	0.044	0.996	−0.002	0.772	−0.002	0.683	−0.103	0.952
**u2**	−0.003	0.831	0.084	0.961	0.052	0.996	0.036	0.981	0.034	0.998	−0.053	0.962
**u3**	−0.032	0.974	0.047	0.969	0.026	1.000	−0.003	0.654	−0.004	0.838	−0.104	0.950
**u4**	−0.025	0.976	0.055	0.967	0.037	0.998	0.036	0.981	0.034	0.999	−0.049	0.965
**u5**	−0.025	0.974	0.056	0.968	0.037	0.998	0.045	0.972	0.041	0.997	−0.039	0.979
**u6**	−0.056	0.968	0.018	0.983	0.010	0.989	0.040	0.974	0.037	0.998	−0.046	0.963

**Table 4:** Energies (in kcal/mol) of intramolecular hydrogen bonds in the gas phase calculated by means of rotational method (energy of **u6** minus **u5**) and from the Afonin equation (Equation (1)).

	Rotational	Afonin
**u6** 5-NO_2_	−7.49	−7.55
**u6** 5-NH_2_	−3.36 *	−2.61

* Calculated by rotating the OH group in the 4 position by 180° with NH_2_ group frozen in **u6** 5-NH_2_ conformation (perpendicular relative to the plane of the ring).

**Table 5:** Energies (in kcal/mol) relative to the **u1** tautomer. Δ indicates a difference in relative energies between the aqueous phase and the gas phase, Δ = *E*_rel_(aq) − *E*_rel_(gas).

Taut.	H	5-NH_2_	Δ	5-NO_2_	Δ	5-NO_2_ (90°)	Δ	6-NH_2_	Δ	6-NO_2_	Δ	6-NO_2_ (90°)
**u1**	0.0	0.0	0.0	0.0	0.0	0.0	0.0	0.0	0.0	0.0	0.0	0.0
**u2**	11.1	9.5	2.7	10.1	2.1	9.1	2.5	6.6	3.8	9.9	1.1	5.4
**u3**	11.4	14.6	−2.2	10.2	1.1	11.8	0.2	10.3	−0.6	11.4	0.4	11.1
**u4**	13.2	14.4	3.6	11.8	5.9	11.6	5.6	8.1	6.5	11.7	1.6	7.2
**u5**	14.2	15.5	2.9	12.9	5.0	12.6	4.8	8.5	6.4	12.9	0.5	8.1
**u6**	17.9	18.1	2.1	5.4	7.3	15.8	4.8	12.4	3.9	16.2	1.3	11.7

**Table 6:** Slopes, *a*, of *E*_rel_ = *a* ∙ (1/ε) + *b* linear regressions (in all cases *R*^2^ > 0.97) and molecular dipole moments in the gas phase, *μ* (*E*_rel_ in kcal/mol, *μ* in Debye).

Tautomer	5-NH_2_		5-NO_2_		5-NO_2_ (90°)		6-NO_2_		6-NO_2_ (90°)		6-NH_2_	
	*a*	*μ*	*a*	*μ*	*a*	*μ*	*a*	*μ*	*a*	*μ*	*a*	*μ*
**u1**	0.0180	4.5	0.0234	4.9	0.0207	4.7	0.0152	0.5	0.0164	1.1	0.0231	6.2
**u2**	0.0135	2.3	0.0200	7.0	0.0168	6.3	0.0170	4.5	0.0155	4.2	0.0168	4.8
**u3**	0.0216	5.9	0.0217	2.2	0.0203	2.3	0.0159	2.8	0.0167	2.6	0.0242	6.6
**u4**	0.0120	2.6	0.0137	3.8	0.0120	2.8	0.0126	3.3	0.0112	2.8	0.0125	3.2
**u5**	0.0133	3.3	0.0152	4.7	0.0132	3.7	0.0144	5.8	0.0125	5.4	0.0127	1.9
**u6**	0.0145	3.8	0.0115	1.8	0.0130	1.3	0.0173	4.7	0.0157	4.5	0.0168	4.2

## Data Availability

The data presented in this study are available in the article and in the associated Supplementary Materials.

## Supplementary Materials

The following supporting information can be downloaded at: https://www.mdpi.com/article/10.3390/molecules27217240/s1, Table S1: Dipole moments; Tables S2–S21: Relative and solvation electronic energies and Gibbs energies of all systems; Tables S22 and S23: Statistical data on *G* and *G*_solv_ vs. 1/ε correlations; Tables S24–S29: cSAR(X) values for nitro and amino substituents in all systems; Table S30: Geometry data in the gas phase and formamide; Table S31: Differences between CN bond length in formamide and the gas phase; Table S32: CO bond lengths of C=O/C-OH groups in 2 and 4 position of the uracil molecule; Table S33: cSAR(X) values of C=O/C-OH groups; Table S34: Ranges and average values of cSAR(X) of C=O/C-OH groups in 2 and 4 position.
