# Supplementary material for: Intramolecular Interactions in Derivatives of Uracil Tautomers
Source: Molecules. 2022 Oct 25;27(21):7240. doi: 10.3390/molecules27217240 (PMC9656941; doi:10.3390/molecules27217240)
Supplement: Supplementary file 1 [file molecules-27-07240-s001.zip › molecules-1964715-supplementary.pdf]

Supplementary Materials

Intramolecular interactions in derivatives of uracil tautomers

Paweł A. Wieczorkiewicz[1], Tadeusz M. Krygowski[2] and Halina Szatylowicz[1]
[1] Faculty of Chemistry, Warsaw University of Technology, Noakowskiego 3, 00-664 Warsaw, Poland
[2] Department of Chemistry, University of Warsaw, Pasteura 1, 02-093 Warsaw, Poland

Table of Contents

Table S1. Dipole moments	2
Table S2. Relative energies of 5-NH2 uracil tautomers in all studied environments	2
Table S3. Solvation energies of 5-NH2 uracil tautomers in all studied environments	2
Table S4. Relative Gibbs energies 5-NH2 uracil tautomers in all studied environments	2
Table S5. Solvation Gibbs energies of 5-NH2 uracil tautomers in all studied environments	2
Table S6. Relative energies of 5-NO2 uracil tautomers in all studied environments	3
Table S7. Solvation energies of 5-NO2 uracil tautomers in all studied environments	3
Table S8. Relative Gibbs energies of 5-NO2 uracil tautomers in all studied environments	3
Table S9. Solvation Gibbs energies of 5-NO2 uracil tautomers in all studied environments	3
Table S10. Relative energies of 5-NO2(90°) uracil tautomers in all studied environments	3
Table S11. Solvation energies of 5-NO2(90°) uracil tautomers in all studied environments	4
Table S12. Relative energies of 6-NH2 uracil tautomers in all studied environments	4
Table S13. Solvation energies of 6-NH2 uracil tautomers in all studied environments	4
Table S14. Relative Gibbs energies 6-NH2 uracil tautomers in all studied environments	4
Table S15. Solvation Gibbs energies of 6-NH2 uracil tautomers in all studied environments	4
Table S16. Relative energies of 6-NO2 uracil tautomers in all studied environments	5
Table S17. Solvation energies of 6-NO2 uracil tautomers in all studied environments	5
Table S18. Relative Gibbs energies of 6-NO2 uracil tautomers in all studied environments	5
Table S19. Solvation Gibbs energies of 6-NO2 uracil tautomers in all studied environments	5
Table S20. Relative energies of 6-NO2(90°) uracil tautomers in all studied environments	5
Table S21. Solvation energies of 6-NO2(90°) uracil tautomers in all studied environments	6
Table S22. Values of a and determination coefficients of G = $a \cdot 1/\varepsilon + b$ correlations, where G is the total Gibbs energy of a molecule (in hartree) and ε is the dielectric constant of the environment	6
Table S23. Values of a and determination coefficients of G_solv = $a \cdot 1/\varepsilon + b$ correlations	6
Table S24. cSAR(X) values for the amino group in 5-NH2 uracil tautomers in all studied environments	6
Table S25. cSAR(X) values for the nitro group in 5-NO2 uracil tautomers in all studied environments	6
Table S26. cSAR(X) values for the nitro group in 5-NO2(90°) uracil tautomers in all studied environments	7
Table S27. cSAR(X) values for the amino group in 6-NH2 uracil tautomers in all studied environments	7
Table S28. cSAR(X) values for the nitro group in 6-NO2 uracil tautomers in all studied environments	7
Table S29. cSAR(X) values for the nitro group in 6-NO2(90°) uracil tautomers in all studied environments	7
Table S30. Geometry data in the gas phase (_g) and formamide (_f), obtained at B97D3/aug-cc-pVDZ level	8
Table S31. Differences between CN bond lengths in formamide and the gas phase	9
Table S32. CO bond lengths of C=O/C-OH groups in 2 and 4 position of the uracil molecule (in the gas phase)	9
Table S33. cSAR(X) values of C=O/C-OH groups in 2 and 4 position of the uracil molecule in the gas phase	9
Table S34. Ranges and average values of cSAR(X) of C=O/C-OH groups in 2 and 4 position of the uracil molecule, in the gas phase	9

Table S1. Dipole moments μ (in Debye) and the difference in μ between formamide and the gas phase, Δμ = μ(f) – μ(gas).

	**5-NH2**		**5-NO2**		**5-NO2(90°)**		**6-NO2**		**6-NO2(90°)**		**6-NH2**	
	μ	Δμ	μ	Δμ	μ	Δμ	μ	Δμ	μ	Δμ	μ	Δμ
**u1**	4.5	1.9	4.9	2.5	4.7	2.0	0.5	0.5	1.1	0.8	6.2	3.2
**u2**	2.3	1.1	7.0	3.2	6.3	2.4	4.5	1.6	4.2	1.5	4.8	2.1
**u3**	5.9	2.6	2.2	1.1	2.3	1.2	2.8	1.3	2.6	1.3	6.6	3.3
**u4**	2.6	1.0	3.8	1.5	2.8	0.7	3.3	1.0	2.8	0.8	3.2	1.4
**u5**	3.3	1.3	4.7	1.7	3.7	1.0	5.8	2.0	5.4	1.7	1.9	0.9
**u6**	3.8	1.9	1.8	0.6	1.3	0.4	4.7	1.8	4.5	1.6	4.2	1.9

Table S2. Relative energies (in kcal·mol^{-1}) of 5-NH2 uracil tautomers in all studied environments.

**E_rel**	**gas**	**to**	**chl**	**o-cr**	**thf**	**pyr**	**et**	**dmso**	**water**	**f**	**range**
**u1**	0.0	0.0	0.0	0.0	0.0	0.0	0.0	0.0	0.0	0.0	0.0
**u2**	9.5	10.7	11.3	11.6	11.6	11.9	12.1	12.2	12.2	12.2	2.7
**u3**	14.6	13.8	13.2	13.0	12.9	12.7	12.5	12.5	12.4	12.4	-2.2
**u4**	14.4	16.1	17.0	17.3	17.4	17.7	17.9	18.0	18.1	18.1	3.6
**u5**	15.5	16.9	17.6	17.8	17.9	18.1	18.3	18.3	18.4	18.4	2.9
**u6**	18.1	19.3	19.8	19.9	20.0	20.1	20.2	20.2	20.3	20.3	2.1

Table S3. Solvation energies (in kcal·mol^{-1}) of 5-NH2 uracil tautomers in all studied environments.

**E_solv**	**gas**	**to**	**chl**	**o-cr**	**thf**	**pyr**	**et**	**dmso**	**water**	**f**	*range*
**u1**	0.0	-5.0	-7.7	-8.7	-8.9	-9.8	-10.5	-10.8	-11.0	-11.1	-11.0
**u2**	0.0	-3.8	-5.8	-6.6	-6.7	-7.4	-7.9	-8.2	-8.3	-8.3	-8.3
**u3**	0.0	-5.9	-9.1	-10.3	-10.6	-11.7	-12.6	-13.0	-13.2	-13.3	-13.2
**u4**	0.0	-3.4	-5.1	-5.8	-5.9	-6.6	-7.0	-7.3	-7.4	-7.4	-7.4
**u5**	0.0	-3.7	-5.7	-6.4	-6.5	-7.3	-7.8	-8.0	-8.2	-8.2	-8.2
**u6**	0.0	-3.8	-6.0	-6.9	-7.0	-7.9	-8.4	-8.7	-8.9	-9.0	-8.9

Table S4. Relative Gibbs energies (in kcal·mol^{-1}) of 5-NH2 uracil tautomers in all studied environments.

**G_rel**	**gas**	**to**	**chl**	**o-cr**	**thf**	**pyr**	**et**	**dmso**	**water**	**f**	*range*
**u1**	0.0	0.0	0.0	0.0	0.0	0.0	0.0	0.0	0.0	0.0	0.0
**u2**	9.4	10.5	11.1	11.4	11.4	11.7	11.8	11.8	11.9	11.9	2.4
**u3**	14.1	13.4	13.0	12.8	12.8	12.6	12.4	12.3	12.2	12.2	-1.9
**u4**	14.3	15.9	16.7	17.0	17.1	17.4	17.6	17.6	17.7	17.7	3.3
**u5**	15.4	16.6	17.2	17.5	17.5	17.7	17.8	17.9	17.9	17.9	2.6
**u6**	17.8	18.5	19.4	19.6	19.7	19.8	19.8	19.9	19.9	19.9	2.1

Table S5. Solvation Gibbs energies (in kcal·mol^{-1}) of 5-NH2 uracil tautomers in all studied environments.

**G_solv**	**gas**	**to**	**chl**	**o-cr**	**thf**	**pyr**	**et**	**dmso**	**water**	**f**	*range*
**u1**	0.0	-5.0	-7.7	-8.7	-8.9	-9.8	-10.4	-10.8	-10.9	-11.0	-10.9
**u2**	0.0	-4.0	-6.0	-6.7	-6.9	-7.6	-8.1	-8.4	-8.5	-8.5	-8.5
**u3**	0.0	-5.7	-8.8	-10.0	-10.2	-11.4	-12.2	-12.6	-12.8	-12.9	-12.8
**u4**	0.0	-3.4	-5.3	-6.0	-6.1	-6.8	-7.2	-7.5	-7.6	-7.6	-7.6
**u5**	0.0	-3.8	-5.8	-6.6	-6.7	-7.4	-8.0	-8.2	-8.4	-8.4	-8.4
**u6**	0.0	-4.3	-6.1	-6.8	-7.0	-7.8	-8.4	-8.7	-8.8	-8.9	-8.8

Table S6. Relative energies (in kcal·mol^{-1}) of 5-NO2 uracil tautomers in all studied environments.

E_rel	gas	to	chl	o-cr	thf	pyr	et	dmso	water	f	*range*
**u1**	0.0	0.0	0.0	0.0	0.0	0.0	0.0	0.0	0.0	0.0	0.0
**u2**	10.1	11.0	11.5	11.7	11.8	11.9	12.0	12.1	12.1	12.1	2.1
**u3**	10.2	10.6	10.9	11.0	11.0	11.1	11.2	11.2	11.2	11.2	1.1
**u4**	11.8	14.4	15.8	16.3	16.4	17.0	17.4	17.6	17.7	17.7	5.9
**u5**	12.9	15.1	16.4	16.8	16.9	17.4	17.7	17.8	17.9	17.9	5.0
**u6**	5.4	8.7	10.5	11.1	11.2	11.9	12.3	12.6	12.7	12.7	7.3

Table S7. Solvation energies (in kcal·mol^{-1}) of 5-NO2 uracil tautomers in all studied environments.

E_solv	gas	to	chl	o-cr	thf	pyr	et	dmso	water	f	*range*
**u1**	0.0	-6.4	-9.9	-11.2	-11.4	-12.7	-13.6	-14.1	-14.3	-14.4	-14.3
**u2**	0.0	-5.5	-8.5	-9.6	-9.7	-10.9	-11.7	-12.1	-12.3	-12.3	-12.3
**u3**	0.0	-5.9	-9.2	-10.4	-10.6	-11.8	-12.6	-13.1	-13.3	-13.4	-13.3
**u4**	0.0	-3.8	-5.9	-6.6	-6.8	-7.5	-8.0	-8.3	-8.4	-8.5	-8.4
**u5**	0.0	-4.1	-6.4	-7.3	-7.4	-8.3	-8.8	-9.2	-9.3	-9.4	-9.3
**u6**	0.0	-3.1	-4.8	-5.5	-5.6	-6.2	-6.7	-6.9	-7.0	-7.1	-7.0

Table S8. Relative Gibbs energies (in kcal·mol^{-1}) of 5-NO2 uracil tautomers in all studied environments.

G_rel	gas	to	chl	o-cr	thf	pyr	et	dmso	water	f	*range*
**u1**	0.0	0.0	0.0	0.0	0.0	0.0	0.0	0.0	0.0	0.0	0.0
**u2**	10.0	10.6	11.2	11.4	11.6	11.7	11.8	11.9	12.0	12.0	2.0
**u3**	9.9	10.3	10.6	10.7	11.3	10.9	11.0	11.1	11.1	11.1	1.4
**u4**	11.5	13.6	15.2	15.8	16.5	16.5	17.0	17.2	17.3	17.4	5.8
**u5**	12.7	14.3	15.7	16.2	16.9	16.9	17.2	17.4	17.5	17.6	4.8
**u6**	6.4	9.3	11.0	11.6	12.3	12.4	12.9	13.1	13.3	13.3	6.9

Table S9. Solvation Gibbs energies (in kcal·mol^{-1}) of 5-NO2 uracil tautomers in all studied environments.

G_solv	gas	to	chl	o-cr	thf	pyr	et	dmso	water	f	*range*
**u1**	0.0	-6.2	-9.6	-10.9	-11.7	-12.5	-13.4	-13.9	-14.2	-14.3	14.3
**u2**	0.0	-5.6	-8.4	-9.5	-10.2	-10.8	-11.6	-12.0	-12.2	-12.3	12.3
**u3**	0.0	-5.8	-9.0	-10.1	-10.4	-11.5	-12.4	-12.8	-13.0	-13.1	13.1
**u4**	0.0	-4.1	-5.9	-6.6	-6.8	-7.5	-8.0	-8.3	-8.4	-8.4	8.4
**u5**	0.0	-4.6	-6.6	-7.4	-7.6	-8.4	-8.9	-9.2	-9.4	-9.4	9.4
**u6**	0.0	-3.2	-5.0	-5.7	-5.8	-6.5	-6.9	-7.2	-7.3	-7.3	7.3

Table S10. Relative energies (in kcal·mol^{-1}) of 5-NO2(90°) uracil tautomers in all studied environments.

E_rel	gas	thf	water	*range*
**u1**	0.0	0.0	0.0	0.0
**u2**	9.1	11.0	11.6	2.5
**u3**	11.8	12.0	12.0	0.2
**u4**	11.6	16.0	17.1	5.6
**u5**	12.6	16.5	17.4	4.8
**u6**	15.8	19.7	20.6	4.8

Table S11. Solvation energies (in kcal·mol^{-1}) of 5-NO2(90°) uracil tautomers in all studied environments.

E_solv	gas	thf	water	*range*
**u1**	0.0	-10.6	-13.2	13.2
**u2**	0.0	-8.7	-10.7	10.7
**u3**	0.0	-10.4	-13.0	13.0
**u4**	0.0	-6.2	-7.6	7.6
**u5**	0.0	-6.8	-8.4	8.4
**u6**	0.0	-6.7	-8.4	8.4

Table S12. Relative energies (in kcal·mol^{-1}) of 6-NH2 uracil tautomers in all studied environments.

E_rel	gas	to	chl	o-cr	thf	pyr	et	dmso	water	f	*range*
**u1**	0.0	0.0	0.0	0.0	0.0	0.0	0.0	0.0	0.0	0.0	0.0
**u2**	6.6	8.3	9.3	9.6	9.7	10.0	10.3	10.4	10.5	10.5	3.8
**u3**	10.3	10.2	10.0	9.9	9.9	9.8	9.7	9.7	9.7	9.7	-0.6
**u4**	8.1	11.1	12.7	13.3	13.4	14.0	14.4	14.6	14.7	14.7	6.5
**u5**	8.5	11.4	12.9	13.5	13.6	14.2	14.5	14.7	14.8	14.9	6.4
**u6**	12.4	14.4	15.3	15.6	15.6	15.9	16.1	16.2	16.3	16.3	3.9

Table S13. Solvation energies (in kcal·mol^{-1}) of 6-NH2 uracil tautomers in all studied environments.

E_solv	gas	to	chl	o-cr	thf	pyr	et	dmso	water	f	*range*
**u1**	0.0	-6.5	-9.9	-11.2	-11.4	-12.7	-13.5	-14.0	-14.2	-14.2	-14.2
**u2**	0.0	-4.8	-7.3	-8.2	-8.4	-9.3	-9.9	-10.2	-10.3	-10.4	-10.3
**u3**	0.0	-6.7	-10.3	-11.6	-11.9	-13.2	-14.1	-14.6	-14.8	-14.9	-14.8
**u4**	0.0	-3.5	-5.4	-6.0	-6.2	-6.8	-7.3	-7.5	-7.6	-7.7	-7.6
**u5**	0.0	-3.6	-5.5	-6.1	-6.3	-7.0	-7.4	-7.7	-7.8	-7.9	-7.8
**u6**	0.0	-4.6	-7.1	-8.0	-8.2	-9.2	-9.8	-10.1	-10.3	-10.4	-10.3

Table S14. Relative Gibbs energies (in kcal·mol^{-1}) of 6-NH2 uracil tautomers in all studied environments.

G_rel	gas	to	chl	o-cr	thf	pyr	et	dmso	water	f	*range*
**u1**	0.0	0.0	0.0	0.0	0.0	0.0	0.0	0.0	0.0	0.0	0.0
**u2**	6.6	8.1	9.2	9.5	9.6	9.9	10.1	10.3	10.4	10.4	3.8
**u3**	10.1	9.9	9.8	9.7	9.7	9.5	9.4	9.4	9.3	9.3	0.8
**u4**	8.4	11.1	12.8	13.3	13.4	13.9	14.3	14.5	14.6	14.7	6.2
**u5**	8.7	11.4	13.0	13.5	13.6	14.1	14.5	14.7	14.8	14.8	6.2
**u6**	12.5	14.4	15.4	15.6	15.7	15.9	16.1	16.2	16.2	16.2	3.7

Table S15. Solvation Gibbs energies (in kcal·mol^{-1}) of 6-NH2 uracil tautomers in all studied environments.

G_solv	gas	to	chl	o-cr	thf	pyr	et	dmso	water	f	*range*
**u1**	0.0	-6.5	-10.1	-11.3	-11.6	-12.8	-13.7	-14.2	-14.4	-14.5	14.5
**u2**	0.0	-5.0	-7.5	-8.5	-8.7	-9.6	-10.2	-10.5	-10.6	-10.7	10.7
**u3**	0.0	-6.7	-10.4	-11.7	-12.0	-13.4	-14.4	-14.9	-15.2	-15.3	15.3
**u4**	0.0	-3.8	-5.8	-6.5	-6.6	-7.3	-7.8	-8.1	-8.2	-8.2	8.2
**u5**	0.0	-3.8	-5.8	-6.5	-6.7	-7.4	-7.9	-8.1	-8.3	-8.3	8.3
**u6**	0.0	-4.6	-7.2	-8.2	-8.4	-9.4	-10.1	-10.5	-10.7	-10.7	10.7

Table S16. Relative energies (in kcal·mol^{-1}) of 6-NO2 uracil tautomers in all studied environments.

E_rel	gas	to	chl	o-cr	thf	pyr	et	dmso	water	f	*range*
**u1**	0.0	0.0	0.0	0.0	0.0	0.0	0.0	0.0	0.0	0.0	0.0
**u2**	9.9	9.3	9.1	9.0	9.0	8.9	8.8	8.8	8.8	8.8	1.1
**u3**	11.4	11.3	11.2	11.2	11.1	11.1	11.0	11.0	11.0	11.0	0.4
**u4**	11.7	12.4	12.8	13.0	13.0	13.2	13.3	13.3	13.3	13.3	1.6
**u5**	12.9	13.3	13.4	13.4	13.4	13.4	13.4	13.4	13.4	13.4	0.5
**u6**	16.2	15.9	15.5	15.4	15.4	15.2	15.1	15.0	15.0	15.0	1.3

Table S17. Solvation energies (in kcal·mol^{-1}) of 6-NO2 uracil tautomers in all studied environments.

E_solv	gas	to	chl	o-cr	thf	pyr	et	dmso	water	f	*range*
**u1**	0.0	-4.3	-6.5	-7.4	-7.5	-8.3	-8.9	-9.2	-9.3	-9.4	9.4
**u2**	0.0	-4.9	-7.4	-8.3	-8.5	-9.3	-9.9	-10.3	-10.4	-10.5	10.5
**u3**	0.0	-4.4	-6.8	-7.6	-7.8	-8.7	-9.3	-9.6	-9.7	-9.8	9.8
**u4**	0.0	-3.6	-5.4	-6.1	-6.2	-6.9	-7.4	-7.6	-7.7	-7.8	7.8
**u5**	0.0	-4.0	-6.1	-6.9	-7.1	-7.9	-8.4	-8.7	-8.8	-8.9	8.9
**u6**	0.0	-4.6	-7.2	-8.2	-8.4	-9.4	-10.0	-10.4	-10.6	-10.6	10.6

Table S18. Relative Gibbs energies (in kcal·mol^{-1}) of 6-NO2 uracil tautomers in all studied environments.

G_rel	gas	to	chl	o-cr	thf	pyr	et	dmso	water	f	*range*
**u1**	0.0	0.0	0.0	0.0	0.0	0.0	0.0	0.0	0.0	0.0	0.0
**u2**	9.2	8.9	8.6	8.6	8.5	8.5	8.4	8.4	8.4	8.4	0.8
**u3**	11.2	11.1	11.0	10.9	10.9	10.9	10.9	10.8	10.8	10.8	0.3
**u4**	11.3	12.2	12.6	12.7	12.8	12.9	13.0	13.0	13.1	13.1	1.7
**u5**	12.3	12.9	13.0	13.1	13.1	13.1	13.1	13.1	13.1	13.1	0.8
**u6**	15.7	15.3	15.0	14.9	14.8	14.7	14.7	14.7	14.7	14.6	1.0

Table S19. Solvation Gibbs energies (in kcal·mol^{-1}) of 6-NO2 uracil tautomers in all studied environments.

G_solv	gas	to	chl	o-cr	thf	pyr	et	dmso	water	f	*range*
**u1**	0.0	-4.3	-6.6	-7.4	-7.6	-8.4	-9.0	-9.3	-9.5	-9.6	9.6
**u2**	0.0	-4.6	-7.2	-8.1	-8.3	-9.2	-9.8	-10.2	-10.3	-10.4	10.4
**u3**	0.0	-4.4	-6.8	-7.6	-7.8	-8.7	-9.3	-9.7	-9.8	-9.9	9.9
**u4**	0.0	-3.4	-5.3	-6.0	-6.2	-6.9	-7.4	-7.6	-7.8	-7.8	7.8
**u5**	0.0	-3.6	-5.8	-6.6	-6.8	-7.6	-8.2	-8.5	-8.7	-8.7	8.7
**u6**	0.0	-4.7	-7.3	-8.2	-8.4	-9.4	-10.0	-10.4	-10.5	-10.6	10.6

Table S20. Relative energies (in kcal·mol^{-1}) of 6-NO2(90°) uracil tautomers in all studied environments.

E_rel	gas	thf	water	*range*
**u1**	0.0	0.0	0.0	0.0
**u2**	5.4	5.8	6.0	0.6
**u3**	11.1	11.0	10.9	0.2
**u4**	7.2	9.9	10.6	3.4
**u5**	8.1	10.1	10.6	2.5
**u6**	11.7	12.2	12.2	0.5

Table S21. Solvation energies (in kcal·mol[-1]) of 6-NO2(90°) uracil tautomers in all studied environments.

E_solv	gas	thf	water	*range*
**u1**	0.0	-8.5	-10.5	10.5
**u2**	0.0	-8.0	-9.9	9.9
**u3**	0.0	-8.6	-10.7	10.7
**u4**	0.0	-5.8	-7.1	7.1
**u5**	0.0	-6.4	-8.0	8.0
**u6**	0.0	-8.0	-10.0	10.0

Table S22. Values of *a* and determination coefficients of G = *a*·1/ε + *b* correlations, where G is the total Gibbs energy of a molecule (in hartree) and ε is the dielectric constant of the environment.

	**5-NH2**		**5-NO2**		**6-NO2**		**6-NH2**	
	a	R^2	*a*	R^2	*a*	R^2	*a*	R^2
**u1**	0.0178	0.980	0.0232	0.971	0.0155	0.975	0.0235	0.977
**u2**	0.0138	0.981	0.0199	0.976	0.0169	0.975	0.0174	0.981
**u3**	0.0209	0.973	0.0212	0.973	0.0160	0.973	0.0247	0.972
**u4**	0.0124	0.976	0.0136	0.984	0.0127	0.971	0.0134	0.979
**u5**	0.0136	0.977	0.0152	0.985	0.0142	0.963	0.0135	0.978
**u6**	0.0142	0.978	0.0119	0.973	0.0172	0.974	0.0174	0.968

Table S23. Values of *a* and determination coefficients of G_solv(kcal·mol[-1]) = *a*·1/ε + *b* correlations.

	**5-NH2**		**5-NO2**		**6-NO2**		**6-NH2**	
	a	R^2	*a*	R^2	*a*	R^2	*a*	R^2
**u1**	11.1942	0.9796	14.5484	0.9713	9.7176	0.9753	14.7345	0.9771
**u2**	8.6785	0.9807	12.4949	0.9763	10.5896	0.9746	10.8955	0.9809
**u3**	13.1180	0.9735	13.3143	0.9732	10.0560	0.9728	15.5109	0.9718
**u4**	7.7720	0.9765	8.5240	0.9839	7.9690	0.9708	8.3812	0.9793
**u5**	8.5509	0.9768	9.5250	0.9847	8.9034	0.9628	8.4624	0.9779
**u6**	8.9180	0.9783	7.4651	0.9727	10.7867	0.9745	10.9279	0.9677

Table S24. cSAR(X) values for the amino group in 5-NH2 uracil tautomers in all studied environments.

tautomer	gas	to	chl	o-cr	thf	pyr	et	dmso	water	f
**u1**	0.067	0.073	0.076	0.076	0.076	0.077	0.078	0.078	0.078	0.078
**u2**	0.078	0.081	0.082	0.082	0.082	0.082	0.082	0.082	0.081	0.081
**u3**	0.057	0.072	0.080	0.083	0.083	0.086	0.088	0.089	0.089	0.089
**u4**	0.068	0.080	0.086	0.088	0.089	0.091	0.092	0.093	0.093	0.093
**u5**	0.068	0.079	0.085	0.088	0.088	0.090	0.092	0.092	0.093	0.093
**u6**	0.042	0.067	0.080	0.085	0.086	0.091	0.094	0.096	0.097	0.097

Table S25. cSAR(X) values for the nitro group in 5-NO2 uracil tautomers in all studied environments.

tautomer	gas	to	chl	o-cr	thf	pyr	et	dmso	water	f
**u1**	-0.163	-0.193	-0.210	-0.217	-0.218	-0.225	-0.229	-0.232	-0.233	-0.234
**u2**	-0.166	-0.202	-0.222	-0.230	-0.234	-0.239	-0.244	-0.247	-0.249	-0.249
**u3**	-0.180	-0.202	-0.213	-0.217	-0.218	-0.222	-0.225	-0.226	-0.227	-0.227
**u4**	-0.173	-0.197	-0.210	-0.215	-0.216	-0.221	-0.225	-0.226	-0.227	-0.228
**u5**	-0.172	-0.198	-0.211	-0.216	-0.217	-0.222	-0.225	-0.227	-0.228	-0.228
**u6**	-0.136	-0.144	-0.149	-0.150	-0.150	-0.152	-0.153	-0.153	-0.153	-0.153

Table S26. cSAR(X) values for the nitro group in 5-NO2(90°) uracil tautomers in all studied environments.

tautomer	gas	thf	water	range
**u1**	-0.121	-0.157	-0.166	-0.045
**u2**	-0.127	-0.170	-0.180	-0.053
**u3**	-0.136	-0.158	-0.162	-0.026
**u4**	-0.138	-0.169	-0.175	-0.037
**u5**	-0.139	-0.169	-0.175	-0.037
**u6**	-0.157	-0.167	-0.167	-0.009

Table S27. cSAR(X) values for the amino group in 6-NH2 uracil tautomers in all studied environments.

	gas	to	chl	o-cr	thf	pyr	et	dmso	water	f
**u1**	0.215	0.258	0.283	0.293	0.295	0.305	0.312	0.316	0.317	0.318
**u2**	0.201	0.224	0.237	0.242	0.243	0.248	0.251	0.253	0.254	0.254
**u3**	0.234	0.277	0.302	0.312	0.314	0.324	0.331	0.335	0.337	0.338
**u4**	0.206	0.227	0.239	0.243	0.244	0.249	0.252	0.253	0.254	0.254
**u5**	0.214	0.232	0.241	0.245	0.245	0.248	0.250	0.252	0.252	0.252
**u6**	0.208	0.229	0.240	0.244	0.245	0.249	0.252	0.253	0.254	0.254

Table S28. cSAR(X) values for the nitro group in 6-NO2 uracil tautomers in all studied environments.

	gas	to	chl	o-cr	thf	pyr	et	dmso	water	f
**u1**	-0.001	0.000	0.000	0.001	0.001	0.001	0.001	0.002	0.002	0.002
**u2**	-0.052	-0.069	-0.078	-0.081	-0.081	-0.084	-0.086	-0.087	-0.087	-0.088
**u3**	0.004	0.004	0.005	0.005	0.005	0.006	0.006	0.006	0.006	0.006
**u4**	-0.042	-0.060	-0.068	-0.071	-0.072	-0.074	-0.076	-0.077	-0.078	-0.078
**u5**	-0.035	-0.056	-0.066	-0.070	-0.071	-0.075	-0.077	-0.079	-0.079	-0.080
**u6**	-0.036	-0.055	-0.065	-0.068	-0.069	-0.072	-0.074	-0.076	-0.076	-0.076

Table S29. cSAR(X) values for the nitro group in 6-NO2(90°) uracil tautomers in all studied environments.

	gas	thf	water
**u1**	0.001	0.002	0.004
**u2**	-0.030	-0.059	-0.065
**u3**	0.013	0.015	0.017
**u4**	-0.021	-0.049	-0.055
**u5**	-0.015	-0.049	-0.057
**u6**	-0.018	-0.049	-0.055

Table S30. Geometry data in the gas phase (_g) and formamide (_f), obtained at B97D3/aug-cc-pVDZ level (all distances in Å). In ortho1 and ortho2 columns the lengths of the closest *ortho* contacts of H or O atoms of NH_2 or NO_2 substituents are given. Ortho1 contact is in the direction of lower atom indexing in the uracil (for example 4 for the 5 substitution), while ortho2 higher (6 for the 5 substitution). dNH1 and dNO1 is the appropriate NH or NO bond length.

	**5-NH2**					**5-NO2**				
	dCN	dNH1	dNH2	ortho1	ortho2	dCN	dNO1	dNO2	ortho1	ortho2
**u1**_g	1.3969	1.0188	1.0149	2.3202	2.6802	1.4671	1.2280	1.2418	2.8197	2.3662
**u2**_g	1.3920	1.0149	1.0141	2.3792	2.6025	1.4674	1.2305	1.2395	2.8636	2.4459
**u3**_g	1.4079	1.0165	1.0166	2.3723	2.6111	1.4639	1.2307	1.2416	2.6874	2.3402
**u4**_g	1.4028	1.0160	1.0161	2.4429	2.5406	1.4666	1.2325	1.2389	2.6986	2.3849
**u5**_g	1.4030	1.0160	1.0161	2.4525	2.5294	1.4661	1.2330	1.2384	2.7022	2.3965
**u6**_g	1.4405	1.0202	1.0202	2.1013	2.9164	1.4406	1.2633	1.2293	1.6700	2.4633
**u1**_f	1.3989	1.0181	1.0163	2.4131	2.5979	1.4512	1.2364	1.2440	2.7988	2.3795
**u2**_f	1.3953	1.0175	1.0159	2.4440	2.5453	1.4509	1.2383	1.2442	2.8147	2.3957
**u3**_f	1.4041	1.0174	1.0173	2.4444	2.5518	1.4510	1.2370	1.2435	2.6767	2.3698
**u4**_f	1.4000	1.0167	1.0169	2.4701	2.5079	1.4535	1.2379	1.2425	2.6787	2.3750
**u5**_f	1.4002	1.0167	1.0169	2.4751	2.5021	1.4531	1.2380	1.2425	2.6807	2.3735
**u6**_f	1.4217	1.0223	1.0189	2.2289	2.5045	1.4342	1.2632	1.2328	1.6839	2.4658

	**5-NO2 (90°)**					**6-NH2**				
	dCN	dNO1	dNO2	ortho1	ortho2	dCN	dNH1	dNH2	ortho1	ortho2
**u1**_g	1.4783	1.2297	1.2297	2.8990	3.2812	1.3857	1.0143	1.0161	2.4712	2.3787
**u2**_g	1.4782	1.2303	1.2302	2.9204	3.2542	1.3748	1.0113	1.0125	2.5173	2.4269
**u3**_g	1.4794	1.2308	1.2309	2.8152	3.3150	1.3789	1.0134	1.0142	2.5118	2.3733
**u4**_g	1.4767	1.2315	1.2315	2.8422	3.2865	1.3742	1.0114	1.0129	2.5434	2.4508
**u5**_g	1.4765	1.2316	1.2316	2.8417	3.2838	1.3723	1.0110	1.0128	2.5459	2.4405
**u6**_g	1.4749	1.2350	1.2345	2.8676	3.3045	1.3739	1.0112	1.0132	2.5326	2.4377
**u1**_f	1.4754					1.3610	1.0123	1.0127	2.4898	2.3174
**u2**_f	1.4747					1.3633	1.0115	1.0123	2.5064	2.4492
**u3**_f	1.4771					1.3546	1.0116	1.0120	2.5306	2.3481
**u4**_f	1.4737					1.3635	1.0116	1.0125	2.5269	2.4769
**u5**_f	1.4735					1.3637	1.0115	1.0125	2.5223	2.4734
**u6**_f	1.4755					1.3632	1.0114	1.0125	2.5118	2.4730

	**6-NO2**					**6-NO2 (90°)**		
	dCN	dNO1	dNO2	ortho1	ortho2	dCN	dNO1	dNO2
**u1**_g	1.4941	1.2257	1.2358	2.5196	2.2005	1.4907	1.2289	1.2289
**u2**_g	1.5201	1.2321	1.2253	2.4413	2.6386	1.4987	1.2283	1.2282
**u3**_g	1.4934	1.2271	1.2341	2.5406	2.2470	1.4902	1.2287	1.2286
**u4**_g	1.5209	1.2331	1.2237	2.4681	2.6616	1.4986	1.2282	1.2281
**u5**_g	1.5247	1.2338	1.2223	2.4577	2.6627	1.5000	1.2280	1.2279
**u6**_g	1.5249	1.2348	1.2215	2.4279	2.6676	1.5006	1.2281	1.2279
**u1**_f	1.4897	1.2278	1.2349	2.5116	2.2153	1.4917		
**u2**_f	1.5149	1.2332	1.2285	2.4480	2.6407	1.5011		
**u3**_f	1.4879	1.2284	1.2341	2.5406	2.2483	1.4909		
**u4**_f	1.5144	1.2332	1.2277	2.4805	2.6580	1.5007		
**u5**_f	1.5163	1.2333	1.2275	2.4758	2.6576	1.5016		
**u6**_f	1.5170	1.2333	1.2272	2.4571	2.6589	1.5018		

Table S31. Differences between CN bond lengths in formamide and the gas phase, $\Delta d_{CN} = d_{CN}(f) - d_{CN}(gas)$.

Δd_{CN}	5-NH2	5-NO2	5-NO2 (90°)	6-NH2	6-NO2	6-NO2 (90°)
**u1**	0.0020	-0.0158	-0.0030	-0.0247	-0.0044	0.0011
**u2**	0.0033	-0.0165	-0.0035	-0.0115	-0.0051	0.0024
**u3**	-0.0037	-0.0128	-0.0024	-0.0244	-0.0055	0.0007
**u4**	-0.0028	-0.0131	-0.0030	-0.0107	-0.0065	0.0020
**u5**	-0.0028	-0.0130	-0.0030	-0.0087	-0.0084	0.0016
**u6**	-0.0188	-0.0064	0.0006	-0.0107	-0.0079	0.0012

Table S32. CO bond lengths of C=O/C-OH groups in 2 and 4 position of the uracil molecule (in the gas phase).

	5-NH2		5-NO2		5-NO2 (90°)		6-NH2		6-NO2		6-NO2 (90°)	
	**4-CO**	**2-CO**	**4-CO**	**2-CO**	**4-CO**	**2-CO**	**4-CO**	**2-CO**	**4-CO**	**2-CO**	**4-CO**	**2-CO**
**u1**	1.234	1.229	1.218	1.221	1.222	1.222	1.232	1.227	1.226	1.220	1.225	1.221
**u2**	1.236	1.358	1.219	1.342	1.223	1.346	1.233	1.353	1.225	1.345	1.225	1.344
**u3**	1.360	1.229	1.338	1.221	1.345	1.222	1.357	1.228	1.349	1.220	1.349	1.221
**u4**	1.361	1.360	1.337	1.344	1.344	1.347	1.357	1.356	1.347	1.346	1.347	1.346
**u5**	1.362	1.361	1.338	1.345	1.345	1.348	1.358	1.356	1.348	1.347	1.348	1.346
**u6**	1.346	1.354	1.324	1.342	1.350	1.347	1.363	1.355	1.353	1.346	1.354	1.345

Table S33. cSAR(X) values of C=O/C-OH groups in 2 and 4 position of the uracil molecule in the gas phase (=O groups in red, -OH in blue).

	position 4						position 2					
	5-NH2	5-NO2	5-NO2 (90°)	6-NH2	6-NO2	6-NO2 (90°)	5-NH2	6-NH2	5-NO2	6-NO2	5-NO2 (90°)	6-NO2 (90°)
**u1**	-0.145	-0.170	-0.097	-0.112	-0.112	-0.116	-0.141	-0.126	-0.086	-0.089	-0.095	-0.087
**u2**	-0.171	-0.193	-0.110	-0.126	-0.128	-0.132	0.169	0.195	0.243	0.240	0.230	0.243
**u3**	0.144	0.131	0.199	0.182	0.187	0.181	-0.165	-0.153	-0.103	-0.107	-0.116	-0.106
**u4**	0.123	0.121	0.198	0.177	0.180	0.175	0.140	0.157	0.215	0.210	0.199	0.210
**u5**	0.117	0.116	0.192	0.172	0.175	0.170	0.141	0.156	0.217	0.209	0.200	0.210
**u6**	0.100	0.125	0.128	0.178	0.174	0.176	0.169	0.161	0.230	0.214	0.206	0.214

Table S34. Ranges and average values of cSAR(X) of C=O/C-OH groups in 2 and 4 position of the uracil molecule, in the gas phase.

	range	average
C4-OH	0.099	0.159
C2-OH	0.103	0.199
C4=O	0.096	-0.134
C2=O	0.078	-0.115
